# Non-equilibrium plasma prevention of *Schistosoma japonicum* transmission

**DOI:** 10.1038/srep35353

**Published:** 2016-10-14

**Authors:** Xing-Quan Wang, Feng-Peng Wang, Wei Chen, Jun Huang, Kateryna Bazaka, Kostya (Ken) Ostrikov

**Affiliations:** 1 School of Physics and Electronic Information, Institute of Optoelectronic Materials and Technology, Gannan Normal University, Ganzhou 341000, China; 2 School of Chemistry, Physics and Mechanical Engineering, Queensland University of Technology, Brisbane, QLD 4000, Australia; 3 Institute of Health and Biomedical Innovation, Queensland University of Technology, Brisbane, QLD 4000, Australia; 4 Institute for Future Environments, Queensland University of Technology, Brisbane, QLD 4000, Australia; 5 CSIRO−QUT Joint Sustainable Processes and Devices Laboratory, Commonwealth Scientific and Industrial Research Organisation, P.O.Box 218, Lindfield, NSW 2070, Australia

## Abstract

*Schistosoma japonicum* is a widespread human and animal parasite that causes intestinal and hepatosplenic schistosomiasis linked to colon, liver and bladder cancers, and anemia. Estimated 230 million people are currently infected with Schistosoma spp, with 779 million people at risk of contracting the parasite. Infection occurs when a host comes into contact with cercariae, a planktonic larval stage of the parasite, and can be prevented by inactivating the larvae, commonly by chemical treatment. We investigated the use of physical non-equilibrium plasma generated at atmospheric pressure using custom-made dielectric barrier discharge reactor to kill *S. japonicum* cercariae. Survival rate decreased with treatment time and applied power. Plasmas generated in O_2_ and air gas discharges were more effective in killing *S. japonicum* cercariae than that generated in He, which is directly related to the mechanism by which cercariae are inactivated. Reactive oxygen species, such as O atoms, abundant in O_2_ plasma and NO in air plasma play a major role in killing of *S. japonicum* cercariae via oxidation mechanisms. Similar level of efficacy is also shown for a gliding arc discharge plasma jet generated in ambient air, a system that may be more appropriate for scale-up and integration into existing water treatment processes.

Schistosomiasis is a significant parasitic disease cause by members of Schistosoma spp. It is estimated that over 230 million people are currently infected with Schistosoma spp^1^, with further 779 million people at risk of contracting the parasites^2^. In China alone, more than 30 million of people are currently at risk of being infected by these trematode flukes, with S. japonicum being the responsible species in Asia, particularly in the Philippines and China.

Once contracted, adult schistosome worms colonise host blood vessels, and are able to effectively evade the immune defense system for years. During this time, they are able to excrete hundreds to thousands of eggs daily^3^. These eggs can exit the body in excreta, contributing to the spread of the parasite within the community, or remain trapped within host tissues, leading to a range of chronic infections and associated diseases. Deleterious local and systemic effects include hepatosplenic disease, urogenital inflammation, periportal fibrosis with portal hypertension, and associated scarring and increased incidence of cancer^1^,^4^. Non-specific morbidities including anaemia, physical effects, such as stunting of growth and reduced physical fitness, and mental effects, such as impaired cognition are also of considerable public health importance, particularly in impoverished communities^5^,^6^,^7^. Notably, schistosomiasis is associated with substantial residual morbidity in a post-infection stage, which brings the number of people currently suffering from the parasite to 440 million^1^,^8^.

Given substantial socioeconomic impacts of the infection in endemic regions, efforts are directed to prevent morbidity, commonly through annual or bi-annual administration of praziquantel^1^, and abolish transmission of the pathogen, by either treatment of infected humans so their excreta are free from pathogen eggs, or by direct treatment of contaminated sewage, or by treatment of contaminated freshwater sources and chemical mollusciciding (to remove intermediate host, the snails). However, many of these efforts are hindered by the lack of precise, sensitive diagnostics of the pathogen^9^. Furthermore, many of these control options lead to significant environmental pollution and ecological damage, or emergence of resistance of the pathogen to schistosomal drugs.

Since infection occurs when an animal or human comes into contact with cercariae, a planktonic larval stage of the parasite, killing the parasite at the cercariae stage of its lifecycle should significantly reduce the incidence of infection. Owing to the fact that over 98% of *S. japonicum* cercariae usually float on the surface of water, chemical treatment of air−water interface is frequently used^10^,^11^,^12^,^13^,^14^. Several plant-derived biocides, including garlic extract solution^10^ and Jatropha seed oils^11^, and exogenous NO^12^ have shown suitable killing efficiency against *S. japonicum* cercaria. Pesticides, such as niclosamide derivatives designed to float on the water surface by decorating a niclosamide core with polyethylene glycol groups of differing chain lengths were able to kill *S. japonicum* cercariae when the number of hydrophilic groups was more than 3^13^. A controlled release strategy based on supramolecular hydrogel of amino acid derivatives, riboflavin, and melamine showed excellent uptake and long-term release of niclosamide derivatives, and high efficiency against cercariae under aqueous conditions^14^. The major limitation with using drug-based approach is the quantity of the chemical required to treat large expanses of water, and associated economic, environmental, and health impacts of such a treatment. It has been reported that UV radiation has damaging effect on cercariae of *Schistosoma mansoni* and *S. haematobium*, with higher treatment doses resulting in decreased cercariae survival, infectivity and maturation. However, it is important to note that for practical application, the use of UV is limited by the finite efficiency, short lamp life, heavy solution absorbance, and the potential for photo-reactivation repair of bacteria^15^,^16^,^17^. Therefore, methods that are more environment- and health-friendly are active sought, particularly for the treatment of drinking water in areas where schistosomiasis is endemic.

In this paper, we investigated physical non-equilibrium plasma as a potential environmentally-benign means to effectively kill *S. japonicum* cercariae. Generated at atmospheric pressure using custom-made dielectric barrier discharge reactor with water as one of the electrodes, plasma delivers a unique and complex mixture of reactive species, including reactive oxygen species (ROS) such as O, O_2_^−^, O_3_ and OH and reactive nitrogen species (RNS) such as NO and NO_2_^18^, electromagnetic radiation, and other effects directly to the surface of the treated object at ambient temperature^19^. Strong oxidative properties of these species make plasma an excellent tool for selective inactivation and physical removal of harmful microorganisms (e.g., bacteria, fungi, spores) on both biotic and temperature-sensitive abiotic surfaces^19^,^20^,^21^,^22^,^23^,^24^,^25^,^26^,^27^,^28^,^29^. The synergistic contributions of electric fields, photons, shockwaves and other physical effects may play an important, but yet-to-be-full elucidated role. High reactivity and relatively short lifespan of the species generated in plasma allows for the treatment to be localized to the surface where cercariae are located^30^, hence minimizing potentially deleterious effects to other aquatic organisms and those who come into contact with thus-treated water^18^. Such spatial and temporal controllability and device scalability make plasma an attractive treatment strategy to explore for the killing of *S. japonicum* cercariae.

## Methods

### Plasma treatment reactor

**Figure 1:**
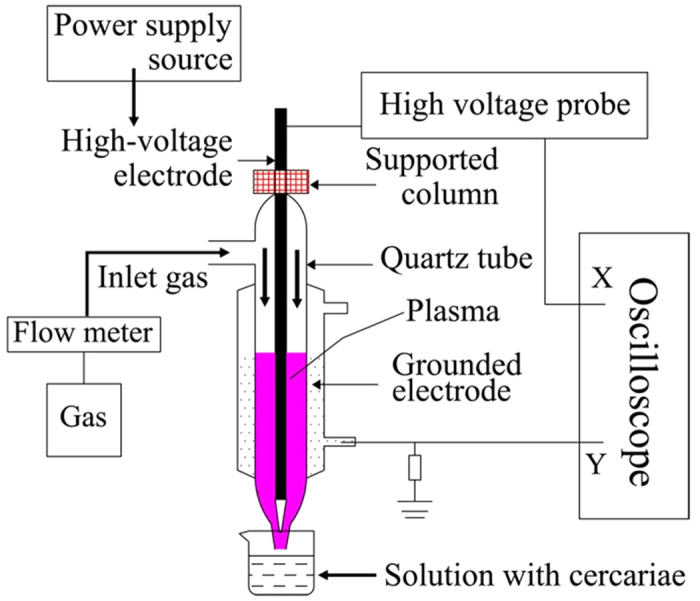
Schematic of the DBD experimental setup.

Experiments were carried out at atmospheric pressure conditions. Plasma was generated using custom-made dielectric barrier discharge (DBD) reactor (Fig. 1), with one electrode being tap water. DBD was generated in a coaxial reactor, in which a quartz tube (inner diameter: 8 mm, outer diameter: 10 mm, length: 200 mm) was used as both a gas feeding tube and the barrier dielectric. A copper bar with a diameter of 5 mm used as the inner high voltage electrode was inserted into the quartz tube by rubber plug fittings. The electrode was connected to an AC power with a maximum peak voltage of 30 kV and an adjustable frequency 6−25 kHz. Tap water in the outer layer of the quartz tube acted as the grounded electrode through a resistor of 50 Ω.

A columnar annular discharge zone (gap 1.5 mm and adjustable length) was formed by the barrier dielectric, the solution electrode and the inner electrode. A plasma jet was generated from the end of the quartz tube, and the length of plasma jet could be controlled by manipulating gas flow rate, applied voltage and frequency. The gas velocity was controlled by the flow meter. Besides helium, gases including argon, nitrogen, oxygen, and air could be used as the working gas, but the plasma jet was more easily generated in helium and argon discharges possibly due to the differences in their ionization energy and metastable living times^31^. To investigate the electric characteristics of discharges, the applied voltages are measured by a P6015A Tektronix HV probe connected to a digital oscilloscope (Tektronix TDS2012, bandwidth: 100 MHz). The current waveforms were obtained by measuring the voltage waveforms on the 50 Ω resistor in series with the discharge loop. The discharge emission spectra were measured by introducing the discharge light into a spectrometer (StellarNet EPP2000, slit width: 25 μm, wavelength range: 190–850 nm). To monitor the changes of pH in the treated water, the precision pH test papers were used.

### Sample preparation and treatment

**Figure 2:**
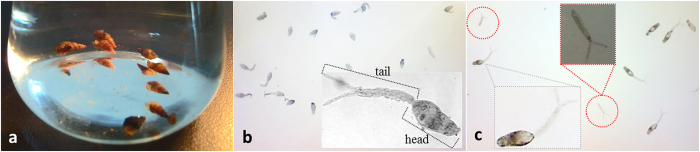
(**a**) Image of intermediate host of *S. japonicum*, the *Oncomelania hupensis* snails. (**b**) Image of *S. japonicum* cercariae released from the snails. c. Image of *S. japonicum* cercariae after treatment show obvious signs of damage, including missing and broken tails (red circles).

The infected intermediate hosts of *Schistosoma japonicum*, small tropical freshwater *Oncomelania hupensis* snails (Fig. 2a) were obtained from Jiangsu Institute of Parasitic Diseases, China. Snails were grown in conical beaker for several hours to release *S. japonicum* cercariae, which would then float on the surface of water. The cercariae were then transferred to 10 ml beakers and visualized using light microscopy (Fig. 2b). Cercariae were measured to be 250−300 μm in length, and displayed morphology typical of healthy organisms.

For plasma treatment, approximately 20 cercariae were placed into each beaker. Prior to treatment, all cercariae were floating on water surface, whereas subsequent to the treatment, dead and damaged organisms (identified by broken or missing tails using light microscopy, Fig. 2c) were shown to promptly sink to the bottom of the flask. The survival rate was estimated as the number of live organisms before and after treatment. The values represent the average (± standard deviation, SD) of a minimum of five independent experiments for each treatment time point and applied power point. The independent t-test was used to determine whether there was a statistically significant difference between the treatment groups.

## Results and Discussion

First, we investigated the characteristics of plasma-assisted killing on cercariae using a dielectric barrier discharge (DBD) reactor. In this experiment, DBD reactor (Fig. 1) was designed to use tap water as one of the electrodes for several reasons. In addition to being abundant and affordable, the use of tap water not only cools down the dielectric efficiently but also avoids the dielectric breakdown due to the different thermal expansions between the metal electrode and the dielectric^32^,^33^,^34^. Second, the solution electrode is in a tight contact with the quartz tube, which makes the electric field distribution more uniform and the plasma more homogeneous, also reduces the power consumption^31^,^35^. Third, the length of solution electrode is adjustable to form different discharge zone by controlling the volume of water used. In this experiment, changing the solution volume was used to adjust the discharge volume and ensure discharge uniformity.

### Effect of gas discharges on *S. japonicum* cercariae

**Figure 3:**
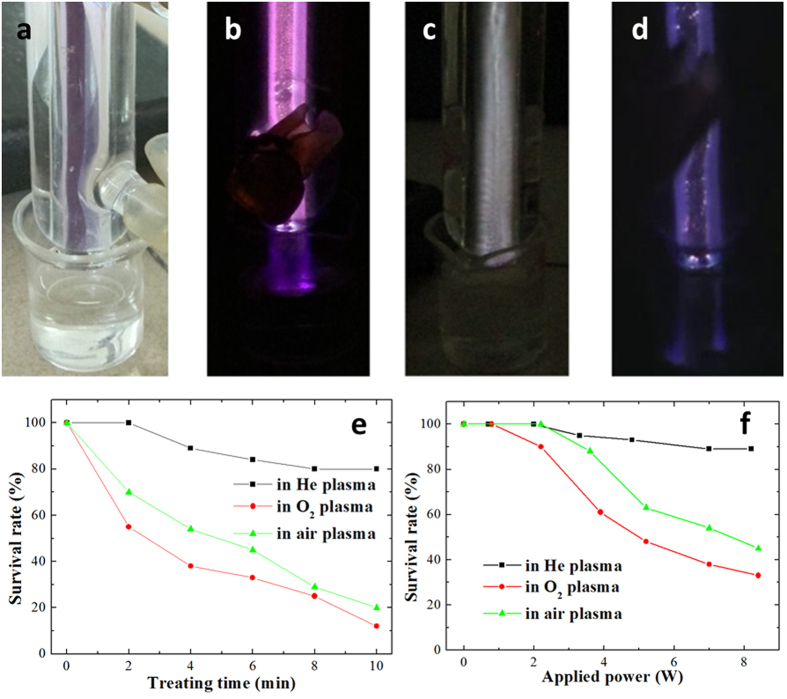
Typical images of discharge treatment on *S. japonicum* cercariae without plasma (**a**) and plasma generated in air (**b**) O_2_ (**c**) He (**d**). Survival curves of *S. japonicum* cercariae in He, O_2_ and air plasmas at a gas flow rate of 100 L/h presented as a function of treatment time (**e**) power of 7 W) and as a function of applied power (**f**) treatment time of 4 min).

In our experiment, we investigated survival curves of *S. japonicum* cercariae after treatment with plasma as a function of the gas type and applied power used to generate plasma, namely He, O_2_ and air gas discharges, and as a function of treatment duration. Figure 3a–d shows typical images of discharges formed in He, O_2_ and air. It can be seen that the discharges are filamentary. The separation between nozzle and solution surface was kept at about 10 mm. It should be noted that for direct plasma treatment, sample drying from gases exiting the nozzle can take place. To control for this effect, solution with cercariae was also treated by the working gases blowing at the same flow rate in the absence of plasma. The results showed that extended exposure (10 min) to gases alone had no statistically significant effect on killing *S. japonicum* cercariae (data not shown).

Figure 3e shows the survival curves of cercariae in He, O_2_ and air plasmas at fixed power and gas flow rate as a function of treatment time. As expected, the survival rate continues to decrease as the duration of the treatment increases, as organisms are exposed to larger doses of reactive chemical species, electromagnetic radiation, and other plasma-generated effects. Amongst the different types of discharges, those produced in He have least killing efficacy, reducing the survival rate to 80%. On the other hand, plasmas produced using O_2_ and air resulted in a substantial decrease in cercariae survival, with the respective survival rates of 12% and 20% after 10 min of plasma treatment. Increasing the intensity of He plasma by increasing the applied power (Fig. 3f) did not substantially change its ability to kill *S. japonicum* cercariae, with the survival rate of 89% at ~8 W after 4 min of treatment. On the contrary, the efficacy with which O_2_ and air plasmas were able to kill *S. japonicum* cercariae increased substantially with applied power, with survival rates decreasing from 85% at ~2 W to 33% at around 8 W in O_2_ plasma, and from 100% at ~2 W to 45% at ~8 W for air plasma. The difference between survival rates in cercariae treated with He, oxygen, and air DBD plasmas for 10 min at 7 W was statistically significant (*p* < 0.05). There was no statistically significant difference in survival rates obtained under similar treatment conditions in repeated experiments, confirming plasma treatment as a reliable method for cercariae inactivation.

### Mechanism of plasma-assisted killing

**Figure 4:**
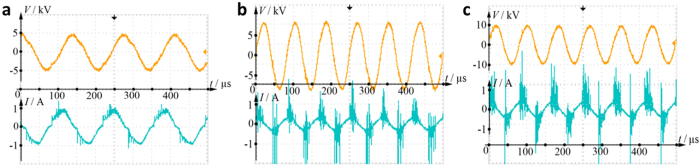
Waveforms of applied voltage and current in (**a**) He, (**b**) O_2_, and (**c**) air gas discharges.

To gain better understanding of the discharges, we investigated the electric characteristics of discharge by measuring the typical waveforms of applied voltage and discharge current in He, O_2_, and air working gases. The voltage−current characteristics of the discharges are shown in Fig. 4. Fig. 4a shows the applied voltage and discharge current in He gas with flow rate of 100 L/h. A sinusoidal resonant power supply was applied to the two electrodes to ignite the discharges in He gas. The working frequency was set at 7 kHz. The voltage−current characteristics confirm that the breakdown of He gas in DBD results in a large number of current filaments called microdischarges, which is in agreement with the image of discharge (Fig. 3d). The microdischarges are randomly distributed both in time and space. The number of microdischarges is proportional to the applied voltage. In this filamentary mode, the discharge starts with local gas breakdown at multiple points within the discharge volume, similar to that observed in plasma needle^21^. This mode is characterized by a periodic current constituted by many discharge pulses in each half cycle. An inverse current peak is also observed when the polarity of the applied voltage changes.

Generated at the flow rate of 100 L/h at a frequency of 12 kHz, the applied voltage and discharge current of O_2_ gas and air discharges are shown in Fig. 4(b,c), respectively. Similar to He discharge, there are numerous current filaments that arise upon application of voltage, however they are stronger and notably denser with the higher breakdown voltage than those observed in He. The filamentary nature of O_2_ and air discharges is in good agreement with the respective images of the discharges. The maximum peak current of about 3 A can be observed in air gas discharge. The electric characteristics of discharge show the electrons frequently run and the frequent collisions that occur among the particles result in the production of high levels of excited reactive species. The presence of these reactive species and the chemistry is confirmed from the emission spectra for these discharges, in which the spectral lines correspond to various active particles^28^,^29^,^32^.

The biological and chemical activity of plasmas is inherently linked to the amount and reactivity of chemical species produced in plasmas, as well as the nature and extent of plasma-generated physical effects, such as photons, electric fields, shock waves, and others^36^,^37^,^38^,^39^. There are a large number of reactive species that can form in atmospheric plasma, such as the high-energy electrons, the excited N atoms, OH radical, N_2_ molecules and O atoms, and the oxygen ions^28^,^32^,^40^,^41^,^42^,^43^. At a sufficient concentration, these species can attack the unsaturated fatty acid component of cell membranes to the extent that cells can no longer maintain membrane integrity and function, eventually leading to cercariae death. It is well known that species such as O and OH are highly reactive, and play an important role in biomedical applications of non-equilibrium atmospheric pressure plasmas^19^,^44^. In addition to attacking the membrane of the cell, O and OH radicals can diffuse across the cell membrane and interact with intracellular components, affecting cell metabolism and functioning, and potentially damaging cell DNA via oxidation^45^,^46^,^47^. The extracellular and intracellular oxidative stress can eventually lead to apoptosis as well as necrosis^48^,^49^,^50^.

**Figure 5:**
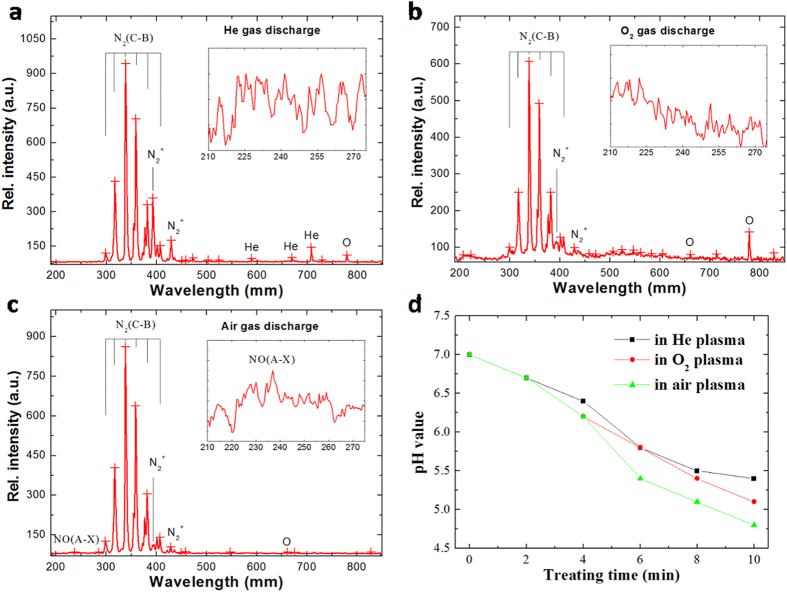
Emission spectra of discharges in He (**a**) O_2_ (**b**) and air (**c**) at a gas flow rate of 100 L/h and a power of 7 W. (**d**) The pH curves in the treated water with He, O_2_ and air plasmas at a gas flow rate of 100 L/h and a power of 7 W presented as a function of treatment time.

To identify the reactive species in plasmas and oxidizing capabilities produced by plasmas, the emission spectra of the produced discharges were measured and respective changes of pH in the treated water were recorded. Figure 5a–c shows the emission spectra of the discharges in He, O_2_ and air. Since the outlet of the discharge tube is open to air, not only He lines but also the lines of atomic O and nitrogen molecules can be seen in the He emission spectrum. It can also be seen that the peaks corresponding to O are very strong in O_2_ emission spectrum. Nitrogen-based species are also evident, however the intensity of the corresponding peaks is significantly lower than that of peaks found in He plasma spectrum. Given the killing efficiency and the intensity of O lines in He and O_2_ discharges, these results suggest that in the case of oxygen plasma treatment, O atoms play a major role in killing *S. japonicum* cercariae. Interestingly, in air discharge, the peak corresponding to atomic O is very weak, suggesting that O atoms are probably not the major species responsible for killing *S. japonicum* cercariae in this type of discharge. On the other hand, a peak for NO is far more prominent in air discharge compared to He and O_2_ discharges. NO exerts its toxic effect by direct nitrosation of DNA and proteins, as well as by combining with reactive oxygen species (such as superoxide and peroxide) and oxidizing the same targets as well as a range of lipids in the cellular membrane^51^,^52^. Therefore, NO might play a major role in killing *S. japonicum* cercariae in air discharge. Given a wide range of biological targets within a living organism with which RNOS species can interact^53^, it may be more difficult for the organisms to develop resistance to plasma treatment.

In addition to emission spectroscopy, changes in the pH values as a result of He, O_2_ and air plasma treatments were measured, as shown in Fig. 5d. All the plasma treatments were performed at power of 7 W. Treatment for 10 min with plasmas resulted in an obvious decrease in the pH value of the solution to 5.4, 5.1 and 4.8 for He, O_2_ and air plasmas, respectively. This was attributed to the effects of nitric and nitrate acids produced from the reaction of H_2_O molecules with NO, which were generated in the gas discharges. In line with the emission data, the pH values of water treated with air discharge was slightly lower due to the higher concentration of nitrogen oxides generated in this type of plasma compared to that generated in He and O_2_ discharges. Given the relatively small difference in pH values between He and O_2_ discharges, the contribution of pH to overall killing efficacy of each type of treatment may not be significant.

To increase the concentration of O and OH radicals, and hence biological activity of the plasma, it is possible to increase the applied power, however it may negatively affect the stability of the discharge. Longer treatment time is another strategy by which higher concentration of species at the cell interface and with the cells of cercariae can be attained. However, from real-life application point of view, prolonged treatment times may not be compatible with current water treatment and decontamination systems and large volumes of water that need to be decontaminated quickly and at low cost. It is also possible to optimize the nature of the processing gas, just as demonstrated in this study where O_2_ and air plasmas were much more efficient in decontaminating water from *S. japonicum* cercariae compared to He plasma (at the same applied power and treatment time).

### Gliding arc plasma jet device

**Figure 6:**
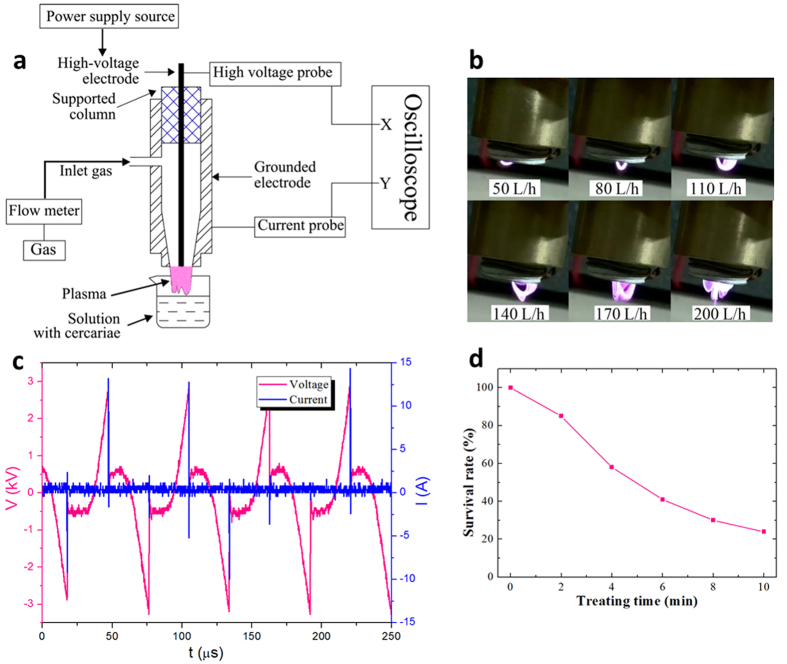
(**a**) Schematic of the GAD plasma jet device. (**b**) Images of discharges generated in GAD plasma jet at different air flow rates and power of 5 W. (**c**) Waveforms of applied voltage and current in GAD. (**d**) Survival curve of *S. japonicum* cercariae in air GAD with different treating times at a gas flow rate of 200 L/h and power of 5 W.

The above results show that atmospheric-pressure plasma is an effective means for killing of *S. japonicum* cercariae. Next, we investigated whether similar level of killing efficacy could be achieved using a different plasma reactor, specifically a gliding arc discharge (GAD) plasma jet generated in ambient air, since this system that may be more appropriate for scale-up and integration into existing water treatment processes compared to DBD, which may be restricted by the gap width. Figure 6a shows the schematic of the GAD plasma jet device. GAD is generated in a coaxial reactor, in which a copper tube (inner diameter: 14 mm, outer diameter: 22 mm, length: 55 mm) with a conic end of inner diameter of 8 mm is used as a gas feeding tube as well as the grounded electrode. In the center of copper tube, a stainless steel needle or tungsten wire with a diameter of 1.6 mm is used as the inner high-voltage electrode by rubber plug fittings. The electrodes are connected to the same power supply as the one used for DBD reactor.

GAD plasma jet is easily generated in air flow driven by the air compressor. When an output voltage of power supply of 4 kV is applied between the central electrode and the copper electrode, an arc will be ignited at the shortest distance and then driven towards the exit of the setup by the flow of gas and electromagnetic force produced by the arc current^54^. Figure 6b shows the formation of GAD plasma jet at different air flow rates and power of 5 W. Since the arc is driven towards the exit of the setup by the flow of air, the area of plasma jet increases with the increasing air flow rate under experimental conditions. To have a better understanding of the arc discharge, the voltage and current of the discharge were recorded with a digital oscilloscope (Fig. 6c) and analyzed. It can be seen that only one current spike at most appear periodically for one voltage peak. The peak current reaches 14.4 A, and is maintained for about 0.1 μs.

Samples of *S. japonicum* cercariae were prepared following the same protocol used in DBD experiments. The separation between nozzle and solution surface was kept at about 10 mm, with a maximum treatment time of 10 min. Time-resolved survival curve of *S. japonicum* cercariae after GAD treatment at a gas flow rate of 200 L/h and power of 5 W is shown in Fig. 6d. Similar to treatment with DBD discharge with air as processing gas, the survival rate of GAD plasma-treated cercariae decrease with treatment time, achieving minimum survival rate of 24% after 10 min of treatment. This result is very similar to that achieved in air discharge generated using DBD reactor, with the difference between survival rates in cercariae treated with air DBD or GAD plasmas for 4 min at 5 W was not statistically significant (*p* > 0.05). This suggests very similar mechanisms of activity via generation of highly reactive oxygen species.

Given the relative ease of scale up, via enlargement of the inner space of copper tube with the conic end or by creating an array of arc discharges, and comparable killing efficacy, GAD-type devices may be more appropriate for integration into real-life water treatment systems compared to DBD-type devices.

## Conclusion

*Schistosoma japonicum* remains a serious disease burden for many developing countries in Asia, associated with long term disabilities that negatively affect health and stunt potential economic development in rural areas. There is a need for novel approaches to limit transmission of this pathogen that does not rely on the use of drugs or potentially toxic chemicals. In this paper, we have demonstrated that non-equilibrium plasma generated at atmospheric pressure can be effectively used to quickly and efficiently kill *S. japonicum* cercariae, the infectious stage of the parasite. We showed that the killing efficacy increases with intensity of plasma and treatment duration, and is also enhanced when working gases rich in oxygen are used. This is possibly due to the fact that oxidative species generated in these plasmas are highly reactive, interfering with several targets on the surface and within the cells, a mechanism that is strongly supported by literature. We have also shown that similar killing efficacy can be attained when discharge is generated using a gliding arc discharge configuration which is more amenable to scale-up. This suggests that the oxygen species-mediated killing mechanisms discussed in this work are generic and may be applicable to other types of plasma devices.

## Additional Information

**How to cite this article**: Wang, X.-Q. *et al.* Non-equilibrium plasma prevention of *Schistosoma japonicum*transmission. *Sci. Rep.* **6**, 35353; doi: 10.1038/srep35353 (2016).
